# Supplementary material for: “It Will Lead You to Make Better Decisions about Your Health”—A Focus Group and Survey Study on Women’s Attitudes towards Risk-Based Breast Cancer Screening and Personalised Risk Assessments
Source: Curr Oncol. 2022 Nov 25;29(12):9181–98. doi: 10.3390/curroncol29120719 (PMC9776908; doi:10.3390/curroncol29120719)
Supplement: Supplementary file 1 [file curroncol-29-00719-s001.zip › Supplementary File S1.pdf]

BREATHE Recruitment Experience Survey
Participant Study ID: ____________

1) How did you get to know of our study? (You may choose more than one option)
   - ☐ Posters at the polyclinic
   - ☐ Posters at the hospital
   - ☐ Pamphlets/Brochures
   - ☐ Referral from family and/or friends
   - ☐ Approached by study coordinator
   - ☐ Others. Please specify ______________

Recruitment Experience

Please help us to understand your **recruitment experience** by selecting which of the following best describes it.

2) The buccal swab was comfortable.
   - ☐ Strongly Agree
   - ☐ Agree
   - ☐ Neither Agree nor Disagree
   - ☐ Disagree
   - ☐ Strongly Disagree

3) The study coordinator explained breast cancer risk classification clearly and is able to answer all of my questions.
   - ☐ Strongly Agree
   - ☐ Agree
   - ☐ Neither Agree nor Disagree
   - ☐ Disagree
   - ☐ Strongly Disagree

4) What was the TOP reason or motivation for joining this study?
   - ☐ To learn about breast cancer
   - ☐ To find out about my risk for breast cancer
   - ☐ To change my screening habits in case I have an increased risk for breast cancer
   - ☐ To inform my children about their possible increased risk for breast cancer
   - ☐ Out of curiosity
   - ☐ Out of scientific/research interest
   - ☐ Others. Please specify: _______________________

CONFIDENTIAL

5) Overall, I am feeling _________ to receive my breast cancer risk report.

	Strongly Agree	Agree	Neither Agree nor Disagree	Disagree	Strongly Disagree
a. Excited					
b. Scared					
c. Confident					
d. Regretful					
e. Optimistic					
f. Anxious					
g. Worried					
h. Stressed out					

i. Others. Please specify: __________________

6) I will recommend doing a breast cancer risk classification to others (family or friends).

- ☐ Strongly Agree
- ☐ Agree
- ☐ Neither Agree nor Disagree
- ☐ Disagree
- ☐ Strongly Disagree

CONFIDENTIAL
