# Supplementary material for: “It Will Lead You to Make Better Decisions about Your Health”—A Focus Group and Survey Study on Women’s Attitudes towards Risk-Based Breast Cancer Screening and Personalised Risk Assessments
Source: Curr Oncol. 2022 Nov 25;29(12):9181–98. doi: 10.3390/curroncol29120719 (PMC9776908; doi:10.3390/curroncol29120719)
Supplement: Supplementary file 1 [file curroncol-29-00719-s001.zip › Supplementary Materials.pdf]

Supplementary Materials

Table S1: Question Guide

1	What is the estimated risk of developing breast cancer in Singaporean women?
2	What are some risk factors related to breast cancer?
3	Do you attend regular health screening for your general health?
4	Do you know of any breast screening programs?
5	What are your breast cancer screening practices? (e.g. breast self-examination/ clinical examination/ mammography)
6	Please tell us about your mammogram experience.
7	Do you plan to have another mammogram in the future? Why/why not?
8	Currently, the national guideline for breast screening is once a year for ages 40 to 49 and once every 2 years for ages 50 and above.<br>Do you adhere to or foresee yourself adhering to such schedules? Why or Why not?
9	Currently, there are people who attend the first screening but very little go for subsequent ones.<br>What are some factors that might have caused that?
10	In your view, what are some suggestions that would make it easier for you to attend mammogram screening more regularly?
11	How can the current breast screening program be improved?
12	Have you talked about breast cancer or breast screening with anyone?
13	How involved is your family when it comes to making decisions with regards to your health?
14	Can you share what influences your decision to receive BCS? (e.g. character, personal beliefs or lifestyle that influence your decision to receive BCS)
15	Analogy: "doctor tells you you have high risk of high blood pressure", would you change lifestyle?<br><br>Doctor tells you you have a high risk of breast cancer. Would you change your lifestyle/attitude?
16	What is your understanding of "genetic risk"?
17	What are the advantages of knowing your personal risk of getting breast cancer and have it as part of your screening result?

18	What are some things that should be noted if genetic risk is included in the screening results?
19	Upon receiving a “low risk” result, how would you feel? (and vice versa)
20	(hypothetical scenario)<br>•Vignette 1: Delia is a 58 year old woman. She is told that her risk of developing breast cancer is lower than average. Delia is therefore advised to decrease her screening frequency from once every 3 years to once every 5 years.<br>• How would you feel if you were Delia?
21	(Brief Conclusions)<br>Is there anything else anyone would want to add?

Table S2: Extrinsic (environmental) factors and quotes on barriers and motivations to mammography

Extrinsic (Environmental) Factors	Quotes
Cost	F35: I think government subsidies on the health screening will encourage more people to go for annual checkups, yeah so subsidise it, maybe less than $10 then maybe people will go.<br><br>F21: I want to go, but maybe cost is a problem, so I might think about going maybe every five years, not every year.
Busy schedules	F4: But in terms of barriers right, sometimes you know life gets in a way it's not something that is at the forefront of my mind.

	F52: I think for me, given the way I've been putting off my own health check-ups right, I might probably fall into the category like what F51 was talking about just now. If everything is fine, you know no family history, feel okay, doctor is always saying yeah you're OK, then I might end up being like too lazy or forgetful.<br><br>F45: Sometimes because busy at work, no time to go.
Availability of information	F24: …it is not being published or we are not well informed of how this should be actually done, so we are not knowledgeable enough to know that there is an easier way for us to get it done.<br><br>F12: I personally feel that Singapore's awareness is still quite low. Like when it's breast screening awareness month, I guess only if you are working in healthcare then you will know. If you are not working in healthcare, there is no big banner saying like "this is breast screening awareness month" that kind of thing.

Company healthcare policies	F48: Yeah I think I will follow the guidelines as well um because I think I'll pay more attention to health, as I grow even older. And plus I would expect that this is part of my insurance, health check-up package, I believe that the mammogram will be part of that package, so I will go for it then.<br><br>F10: So at least the cost is out of the way and you're reminded also. So the work offers something, then you'll always be reminded that "Oh I haven't use it this year." Then you'll go.<br><br>F16: Most of the big companies like MNCs, even though they have annual check-ups, most of the time mammogram won't be included as part of the normal package. So I think it is good if we incorporate that to make it part of the screening package, it will definitely help to reach out to more people.
Presence of reminders	F37: I just recently received a letter from MOH regarding pap smear now, so I think such letters are good to remind people, because without it I also wouldn't know, would not go for it.

	F36: The current apps that we have, like those do with health and then they can flash like according to like your age or something, then you are recommended to do this and there’s a recommendation of location and then know the details, subsidies and stuff.<br><br>F39: Like whenever I go for dental then I’ll book my appointment for the next one, which then I think allows me to constantly go for it. I don't know if there's a such a system set up for breast screening, I think that will be really helpful. They immediately put you in for the next one, and then, when it's near the time they send you a reminder to say, hey you know your screening is scheduled for this day.

Table S3: Intrinsic (personal) factors and quotes on barriers and motivations to mammography

Intrinsic (Personal) Factors	Quotes
Feelings of pain	F53: Yeah so basically when you do it you’re sort of shoved into the small plastic thing, so that they can basically flatten you and then get an accurate picture, but yeah it's kind of painful, but I would rather not.

	F11: I wouldn't mind the waiting time…it's the pain that I really need to get over with it. Because I did it continuously, three times, during my last visit.<br><br>F24: I just went for the mammogram last Saturday so actually I heard from others, that you can be quite painful. So that's one of the reasons why I put it off some time after a few months.
Pessimism towards diagnosis	F40: I think, for some people right which I heard of lah…you don't check, you don't know, nothing happens. The moment you check, *aiya suay suay kenna* [unfortunately detected the cancer], then better don't check. So I think I heard of this a lot of times when I ask some elderly and then they say like "don't want to check, there's nothing now. Check already then I know then it becomes very sad."<br><br>F10: There could also be a fear that you are fine till you go for it…. then you just want to push it aside.

	F13: …people are afraid of finding out any cancerous thing.
Perceived risk	F52: I think for me it’s because I don't feel anything and I have no family history. You know, so I just feel comfortable, although I should I guess you know to just check regularly. I think I'm more aware of these things now that I’ve just reached 40 so yeah, but I don’t know how to check to be honest.<br><br>F15: No particular reason I just haven't felt I need to. I think if it's something like if it's a trigger, like it (breast cancer) happened to our immediate family member, then that would drive me to (screen).
Family history	F2: Oh, I do, yes, on a yearly basis because my father side genetically, my aunt has breast cancer so I'm advised to go for breast screening every year after the age of 40 yeah.
Having a peace of mind	F18: For me, I would. It's just a kind of peace of mind to make sure that everything is okay. And

	this is early detection where I would be able to do something before it's too late.<br><br>F9: I will still go once every two years just to give myself assurance that I'm well.
